# ICD-10 Classification in the Practice of Emergency Medical Teams: New Insights

**DOI:** 10.1155/2024/8506561

**Published:** 2024-05-16

**Authors:** Krzysztof M. Mitura, Jadwiga Snarska, Daniel Celiński, Dominik Maślach, Piotr K. Leszczyński, Aneta Binkowska, Leszek Szpakowski, Sławomir D. Szajda

**Affiliations:** ^1^Independent Public Health Care Center RM-MEDITRANS Emergency Station and Sanitary Transport in Siedlce, Siedlce, Poland; ^2^Department of Surgery, Collegium Medicum University of Warmia and Mazury in Olsztyn, Olsztyn, Poland; ^3^Department of Emergency Medical Service, Medical University of Warsaw, Warsaw, Poland; ^4^Department of Public Health, Medical University of Bialystok, Bialystok, Poland; ^5^Faculty of Medical and Health Sciences, University of Siedlce, Siedlce, Poland; ^6^Department of Emergency Medical Service, Collegium Medicum, University of Warmia and Mazury, Olsztyn, Poland

## Abstract

The role of the emergency medical system is to provide assistance to every person in a state in the event of a sudden threat to health and life. Emergency medical teams (EMTs) are an important element of this system, making diagnoses based on the International Classification of Diseases (ICD-10). The study was aimed at analysing the causes of EMT intervention based on groups of diagnoses codified according to the ICD-10. The analysis was based on data from 116,278 EMT interventions in central-eastern Poland in 2017-2019. The research showed that EMT most often made diagnoses based on groups of ICD-10 codes: R00-R99-Symptoms, signs, and abnormal clinical and laboratory findings, not elsewhere classified (39.11%); S00-T98-Injury, poisoning, and certain other consequences of external causes (18.23%); and I00-I99-Diseases of the circulatory system (15.57%). The analysis of the obtained results showed statistically significant differences (*p* < 0.0001) regarding the area of intervention (urban, rural), sex, age of the patient, and the method of completion of the activities by EMTs in relation to the group of ICD-10 diagnoses for the diagnosis. The conducted study showed the actual reasons for EMT calls. The use of the ICD-10 classification has practical application in EMTs, as it enables the identification of a disease or health problem.

## 1. Introduction

Contemporary emergency medical systems are based on two main organisational models: Anglo-American and French-German [1–4]. The basic assumption of the Anglo-American model is to transport the patient to the hospital as quickly as possible while limiting the number of medical activities at the scene to the necessary, and the basic staff consists of qualified paramedics. The French-German model is based on the presence of a physician in ambulances and the treatment of patients on the spot.

The creation of the Polish State Medical Rescue (SMR) system began in 1999, based on the assumptions of the Anglo-American model [3–5]. The proper shape of the Polish emergency medical system was laid out in 2006 by the Act on the SMR [6]. The SMR system in Poland is aimed at the implementation of the state's tasks consisting of providing assistance to every person who is in a state of sudden health risk, which should be understood as a sudden or predicted appearance of health deterioration symptoms, the direct consequence of which may be serious damage to the body's functions, body injury, or loss of life [6]. The state of a sudden health threat requires immediate medical rescue activities, which are health care services financed from public funds provided by ground emergency medical teams (EMT), air emergency medical teams (AEMT), and hospital emergency departments (HED) [6].

In 2021, there were 1,587 EMTs in Poland, which carried out 3,058,300 interventions, 21 AEMT, and 241 HED [7].

Dispatching EMTs is caused by various reasons reported by witnesses of the event and the patients themselves, but only the arrival at the scene of the event fully verifies the legitimacy of EMT intervention, which ultimately results in making a diagnosis based on the injury, disease entity, observed symptoms, or ailments. The diagnoses made by EMTs are based on the codification according to the International Statistical Classification of Diseases and Related Health Problems (International Classification of Diseases-ICD).

The International Statistical Classification of Diseases, a statistical classification of diseases, originated in the 18th century. The need to introduce a unified classification of causes of death was noted during the first International Statistical Congress in 1853, and in 1881, the International Statistical Institute, which was the successor of the International Statistical Congress, appointed a committee with the French physician, statistician, and demographer Jaques Bertillon (1851-1922) as the head, which commissioned the preparation of a classification of causes of death [8]. Presented in 1893, Bertillon's report, known as the International List of Causes of Death or the Bertillon Classification, was adopted by the International Statistical Congress and, over time, gained international acceptance and use [8]. In turn, during the International Conference on the Revision of the Bertillon Classification of Causes of Death in 1900, a parallel classification of diseases was adopted for use in morbidity statistics [8]. Since its inception, the World Health Organization has been involved in the development of morbidity and mortality classifications based on the Bertillon report [8].

Work on the currently used 10th version of the ICD classification (ICD-10) began in 1983 and ended in 1992 [8]. In Polish health care, ICD-10 has been in force since 1996 [9]. It includes over 14,000 different codes, consisting of 22 chapters covering groups of diseases and health problems. Since January 1, 2022, the 11th version of the International Statistical Classification of Diseases and Health Problems (ICD-11) has been in force. Poland, like other countries, has a 5-year transitional period to introduce this classification.

The use of the ICD-10 codification allows for a statistical presentation of the causes of morbidity and mortality. The use of the ICD-10 codification in emergency medical systems also allows for determining the reasons for intervention and the legitimacy of EMT calls.

The main aim of the study was to analyse the reasons for the intervention of emergency medical teams based on groups of diagnoses codified according to the International Statistical Classification of Diseases and Health Problems ICD-10. The specific objectives included an analysis of the reasons for EMT intervention based on the ICD-10 diagnosis group, depending on the year, call area (urban, rural), patient sex and age, and the method of completion of the intervention.

## 2. Materials and Methods

The analysis concerned the interventions of EMTs in central-eastern Poland for the period from January 1, 2017, to December 31, 2019. The study covered an area of 7,350 km^2^, which at the end of 2019 was inhabited by 547,462 people (women *n* = 277,525; 50.69%, men *n* = 269,937; 49.31%), with the population in urban areas accounting for 30.74% (*n* = 168,314) and in rural areas 69.26% (*n* = 379,148) [10]. An urban area is understood as a city with more than 10,000 inhabitants, and a rural area is understood as an area with less than 10,000 inhabitants. In the analysed period, the Independent Public Healthcare Institution RM-MEDITRANS Ambulance and Sanitary Transport Station in Siedlce (Meditrans) were responsible for the implementation of tasks by EMTs in the field of SMR in the analysed area.

The study was conducted on the basis of a retrospective analysis of data collected in the computer system operating at Meditrans Asseco Medical Management Solutions and derived from medical records kept by EMTs. Due to its nature and the data analysed, the study did not require approval from the Bioethics Committee. Since it did not require human participants, informed consent was not required to participate in the study.

Of all the 122,960 interventions carried out by 23 EMTs registered in the years 2017-2019, 116,278 interventions were ultimately used in the study, excluding 5.43% of calls (*n* = 6,682) due to the lack of data on the patient's sex, age or lack of a patient at the call site.

Detailed data from individual EMT interventions are presented quantitatively based on ICD-10 diagnosis groups depending on the year of the call, intervention area (urban and rural areas), sex, age of the patient, and the method of completion of the intervention by EMTs (transporting the patient to the hospital, leaving summons, death).

The collected data were statistically analysed using the STATISTICA 13.3 program by TIBCO. The relationship between qualitative features was checked using a Chi-square test of independence. The obtained results are presented by number (*n*) and percentage (%). The results were considered statistically significant at *p* < 0.05.

## 3. Results

Throughout the analysed period and in individual years, EMT managers, based on the ICD-10 codification, most often made diagnoses covering the groups of diagnoses R00-R99-symptoms, signs, and abnormal clinical and laboratory findings, not elsewhere classified (total: *n* = 45,471; 39.11%, year 2017: *n* = 14,217; 38.24%, year 2018: *n* = 15,566; 39.60%, year 2019: *n* = 15,688; 39.43%); S00-T98-injury, poisoning, and certain other consequences of external causes (total: *n* = 21,198; 18.23%, year 2017: *n* = 6,981; 18.78%, year 2018: *n* = 7,144; 18.17%, year 2019: *n* = 7,073; 17.77%); and I00-I99-diseases of the circulatory system (total: *n* = 18,110; 15.57%, year 2017: *n* = 5,787; 15.57%, year 2018: *n* = 6,111; 15.55%, year 2018: *n* = 6,212; 15.61%). The least frequent were Q00-Q99-Congenital malformations, deformations, and chromosomal abnormalities (total: *n* = 9; 0.01%, year 2017: *n* = 2; 0.01%, year 2018: *n* = 3; 0.01%, year 2019: *n* = 3; 0.01%); H00-H59-Diseases of the eye and eye appendages (total: *n* = 51; 0.04%, year 2017: *n* = 18; 0.05%, year 2018: *n* = 15; 0.04%, year 2019: *n* = 18; 0.05%); and P00-P96-Certain conditions originating in the perinatal period (total: *n* = 57; 0.05%, year 2017: *n* = 16; 0.04%, year 2018: *n* = 15; 0.04%, year 2019: *n* = 16; 0.07%). There were no diagnoses from the U00-U85-Codes for special purposes. The analysis of the obtained results shows statistically significant differences (*χ*^2^ = 146.32, *p* < 0.0001) between groups of ICD-10 diagnoses and particular years of the analysed period (Table 1).

The conducted analyses show that EMTs were more often dispatched to rural areas (*n* = 77,271; 66.45%) than to urban areas (*n* = 39,007; 33.55%). Both in the case of urban areas and rural areas, EMT calls most often ended with a diagnosis based on the code group R00-R99-Symptoms, signs, and abnormal clinical and laboratory findings, not elsewhere classified (urban-*n* = 14,772; 37.87%, rural-*n* = 30,699; 39.73%); S00-T98-injury, poisoning, and certain other consequences of external causes (city-*n* = 7,351; 18.85%, rural-*n* = 13,847; 17.92%); and I00-I99-Diseases of the circulatory system (urban-*n* = 5,850; 15.00%, rural-*n* = 12,260; 15.87%). Statistically significant differences (*χ*^2^ = 406.73, *p* < 0.0001) were found between groups of diagnoses based on the ICD-10 codification and the area of disposal of EMTs (Table 2).

EMT interventions concerned more men (*n* = 60,678; 52.18%) than women (*n* = 55,600; 47.82%). The most common group of diagnoses in both men and women was the group R00-R99-Symptoms, signs, and abnormal clinical and laboratory findings, not elsewhere classified (*n* = 23,066; 41.49%) compared to men (*n* = 22,405; 36.92%). The next most common groups of diagnoses according to the ICD-10 classification for men were S00-T98-Injury, poisoning, and certain other consequences of external causes (*n* = 12,712; 20.95%) and I00-I99-Diseases of the circulatory system (*n* = 7,524; 12.40%), and in women I00-I99-diseases of the circulatory system (*n* = 10,586; 19.04%) and S00-T98-Injury, poisoning, and certain other consequences of external causes (*n* = 8,486; 15.26%). The analysis of the obtained results indicated statistically significant differences (*χ*^2^ = 3394.57, *p* < 0.0001) between the groups of ICD-10 diagnoses and the patient's sex (Table 3).

In addition, in the study area in 2017-2019, EMTs were most often dispatched to patients aged 70 and over (*n* = 46,074; 39.62%) and aged 50-69 (*n* = 32,291; 27.77%), and least often to people under 15 (*n* = 4,988; 4.29%) and between 15 and 29 (*n* = 11,407; 9.81%). The groups of ICD-10 diagnoses varied depending on the age of the patient. The most common diagnosis concerned the group R00-R99-Symptoms, signs, and abnormal clinical and laboratory findings, not elsewhere classified, in patients 0-14 years old: 36.65% (*n* = 1,828), 30-49 years old: 32.29% (*n* = 6,946), 50-69 years old: 39.61% (*n* = 12,790), and aged 70 and over: 44.65% (*n* = 20,570) of EMT interventions. In the 15-29 age group, the most common diagnosis was S00-T98-Injury, poisoning, and certain other consequences of external causes that constituted 32.43% (*n* = 3,699) of interventions. The most common groups were S00-T98-Injury, poisoning, and certain other consequences of external causes 0-14 years old: 36.55% (*n* = 1,823) and 30-49 years old: 23.91% (*n* = 5,143); I00-I99-Diseases of the circulatory system in the group of patients aged 50-69: 18.30% (*n* = 5,908) and 70 and more years: 23.49% (*n* = 10,824) of interventions and concerning 29.25% (*n* = 3,337) interventions in patients aged 15-29 R00-R99-symptoms, signs, and abnormal clinical and laboratory findings, not elsewhere classified. Statistically significant differences (*χ*^2^ = 23835.29, *p* < 0.0001) were found between the groups of diagnoses based on the ICD-10 codification and the age groups in which EMTs intervened (Table 4).

In the analysed period, EMT activities most often ended with transporting the patient to the hospital (*n* = 86,316; 74.23%), 27,319 interventions ended with leaving the patient at the place of call (23.49%), and in the case of 2,643 trips, medical activities were finally abandoned due to patient death (2.27%). With regard to patients transported to hospitals, the diagnosis was most often made by the EMT manager based on the group of ICD-10 codes R00-R99-Symptoms, signs, and abnormal clinical and laboratory findings, not elsewhere classified (*n* = 34,527; 40.00%); S00-T98-Injury, poisoning, and certain other consequences of external causes (*n* = 18,830; 21.82%); and I00-I99-Diseases of the circulatory system (*n* = 14,087; 16.32%). In the case of leaving the patient at the place of call, the most common diagnoses included R00-R99-symptoms, signs, and abnormal clinical and laboratory findings, not elsewhere classified (*n* = 9,068; 33.19%); I00-I99-Diseases of the circulatory system (*n* = 3,430; 12.56%); and Z00-Z99-Factors influencing health status and contact with health services (*n* = 3,201; 11.72%). In the case of the death of a patient, the most common groups of diagnoses were R00-R99-Symptoms, signs, and abnormal clinical and laboratory findings, not elsewhere classified (*n* = 1,876; 70.98%); I00-I99-Diseases of the circulatory system (*n* = 593; 22.44%), and V01-Y98-External causes of morbidity and mortality (*n* = 115; 4.35%). The analysis of the obtained results indicates statistically significant differences (*χ*^2^ = 15669.11, *p* < 0.0001) regarding the method of completion of activities by EMTs (places of transferring the patient) and the group of ICD-10 diagnoses upon which the diagnosis was made (Table 5).

## 4. Discussion

The ICD-10 classification enables the identification of a disease or health problem and communication of medical personnel. In addition the ICD-10 codification allows for reliable reporting and comparison of medical cases encountered in the prehospital setting and in the entire healthcare industry [11]. The diagnoses made by EMTs are often general and result from limited diagnostic capabilities on board ambulances. They allow action to be taken to help the patient in a life and health emergency. There is no doubt that the diagnoses made at the scene of the call according to the ICD-10 classification are more detailed and clinically relevant than the reasons for the call given by the calling ambulance [11].

In the period studied, the number of EMT interventions amounted to 116,278, which was a higher result than the analysis conducted by Szpakowski et al. in the same area in 2013-2015 [12]. Pittet et al. [13], in a study from 2014, noted the increase in EMT interventions in Europe in the last 20 years, and in the Swiss canton of Vaud from 2001 to 2010; this increase was about 40%. Lowthian et al. [14] point to such factors as the aging of the population as well as the organisation and availability of primary health care as the reason for the increase in the number of EMT interventions.

It was found that the most common diagnoses made by EMTs in the analysed period included diagnoses from the groups: R00-R99-Symptoms, signs, and abnormal clinical and laboratory findings, not elsewhere classified (*n* = 45,471; 39.11%); S00-T98-Injury, poisoning, and certain other consequences of external causes (*n* = 21,198; 18.23%); and I00-I99-diseases of the circulatory system (*n* = 18,110; 15.57%). The conducted research does not allow for a clear indication of the causes of significant differences in the percentage of ICD-10 diagnosis groups in individual years of the analysed period. This is due to too many variables influencing the occurrence and diagnosis of the disease (Table 1). Celiński et al. [15] showed in their research that the most common reasons for EMT calls from Eastern Poland (Biała Podlaska, Chełm) in 2016-2018 to patients aged 65 included the following groups of diagnoses: I00-I99-Diseases of the circulatory system (40.2%); R00-R99-Symptoms, signs, and abnormal clinical and laboratory findings, not elsewhere classified (37.7%); and S00-T98-Injury, poisoning, and certain other consequences of external causes (12.6%). However, the results of studies by Timler et al. on the dispatch of EMTs in 2009 to people over 65 in Otwock county (Poland) indicate that EMTs are most often dispatched to patients with cardiovascular diseases (19%) and injuries (18%) [16]. In the study of EMT interventions in south-eastern Poland in the years 2010-2013, Gawełko and Wilk [17] recorded the highest number of diagnoses for the groups of diagnoses R00-R99-Symptoms, signs, and abnormal clinical and laboratory findings, not elsewhere classified (39%); S00-T98-Injury, poisoning, and certain other consequences of external causes (22%), I00-I99-Diseases of the circulatory system (17%). Moreover, Mitura et al. [18] showed that in the study area in 2015-2018, for calls to workplaces, the most common diagnoses made by EMTs were the following groups of diagnoses: R00-R99-Symptoms, signs, and abnormal clinical and laboratory findings, not elsewhere classified (40.9%); S00-T98-Injury, poisoning, and certain other consequences of external causes (32.4%); and I00-I99-Diseases of the circulatory system (7.9%). However, Cantwell et al. [11], based on data from 2008 to 2011 from the ambulance service in Melbourne, Australia, indicated that the main reasons for EMT interventions were circulatory system disorders (15.60%), injuries and poisonings (13.47%), and diseases of the nervous system (10.37%). The most common diagnoses, according to the ICD-10 codification, performed in emergency services in northern Denmark in the years 2007-2014 were: S00-T98-Injury, poisoning, and certain other consequences of external causes (from 26.3% to 34.0%); R00-R99-Symptoms, signs, and abnormal clinical and laboratory findings, not elsewhere classified (from 14.7% to 28.0%); Z00-Z99-Factors influencing health status and contact with health services (9.6% to 16.5%); and I00-I99-Diseases of the circulatory system (9.5% - 11.5%) [19].

Although ambulance medical staff were competent in diagnosing and treating various diseases [20, 21], as the research shows, imprecise diagnoses were made based on ICD-10 diagnoses from the group R00-R99-Symptoms, signs, and abnormal clinical and laboratory findings, not elsewhere classified. Imprecise diagnoses made by EMTs may result from, among others, the limitations of diagnostics available at the scene and the ambiguity of symptoms [15, 18, 22, 23]. This group of diagnoses includes, among others, diagnoses R07-Pain in throat and chest, R10-Abdominal and pelvic pain, or R55-Syncope and collapse, which may be symptoms in many diseases of various physiological systems, and only wider imaging and laboratory diagnostics performed in hospital conditions allow for a more precise diagnosis.

The most common groups of ICD-10 diagnoses in urban and rural areas include R00-R99-Symptoms, signs, and abnormal clinical and laboratory findings, not elsewhere classified (*n* = 14,772; 37.87% vs. *n* = 30,699; 39.73); S00-T98-Injury, poisoning, and certain other consequences of external causes (*n* = 7,351; 18.85% vs. *n* = 13,847; 17.92%); and I00-I99-Diseases of the circulatory system (*n* = 5,850; 15.00% vs. *n* = 12,260; 15.87%) (Table 2). The analysis of trips based on patient sex reveals that for the most common groups of ICD-10 diagnoses used by EMT managers to diagnose men, the following diagnoses were the most prevalent: S00-T98-injury, poisoning, and certain other consequences of external causes (*n* = 12,712; 20.95%), and in women R00-R99-Symptoms, signs, and abnormal clinical and laboratory findings, not elsewhere classified (*n* = 23,066; 41.49%) and I00-I99-Diseases of the circulatory system (*n* = 10,586; 19.04%) (Table 3). The reasons for EMT intervention depend on a number of factors, including the area of intervention and the patient's sex. Similar correlations regarding the reasons for the intervention, taking into account sex, were also noted by Weiss et al. [24] and in the case of urban and rural areas by Stripe et al. [25] and Aftyka et al. [26]. Thus, factors such as the patient's sex and the area of intervention (urban, rural) significantly determine the operation of EMTs.

EMT interventions for people aged 50 and over constitute the majority (67.39%), with the group of patients between 50 and 74 accounting for 27.77% (*n* = 32,291) and 75 years 39.62% (*n* = 46,074) of all interventions (Table 4). The visible increase in the percentage of trips to the elderly requires a new look at the challenges of the Polish emergency medical system. The aging society in countries such as Great Britain and the United States has forced the creation of new standards of conduct in providing medical rescue services to the elderly on the spot [27, 28].

Apart from EMT interventions, where the diagnosis was made from the group R00-R99-symptoms, signs, and abnormal clinical and laboratory findings, not elsewhere classified, ambulance calls to patients aged 50 and over were caused by cardiovascular diseases. Calls to patients under 50 years old were caused by injuries, external causes of illness, and death, and calls to patients in the 15-59 age group were caused by mental and behavioural disorders (Table 4). These results and the relationship between disease entities and the patient's age were also confirmed by studies conducted in other centres [3, 15–18, 27, 29, 30]. Attention should be paid to the instructions of EMTs to middle-aged patients who were diagnosed in the group F00-F99-Mental and behavioural disorders (Table 4). The current study confirms the studies of other authors about the trend of increasing incidence of mental disorders in Poland and abroad [31, 32].

The significant percentage of EMT interventions resulted in the patient being transported to the hospital (*n* = 86,316; 74.23%), with 23.49% of patients left at the call site (*n* = 27,319) and 2.27% of interventions where EMT activities resulted from patient deaths (*n* = 2,643) (Table 5). Filip et al. [29] reported that in the period they studied, 68.55% of EMT orders resulted in the transport of patients to hospitals, and 18.75% of EMT orders resulted in providing assistance at the scene of the event.

The method of completion of the intervention by EMTs is related to the ICD-10 group on the basis of which the diagnosis was made. However, in the case of the patient's death (*n* = 1,876; 70.98%), the most frequently used were poorly detailed diagnoses from the group of symptoms and disease features (R00-R99-Symptoms, signs, and abnormal clinical and laboratory findings not elsewhere classified) (Table 5).

Most of the diagnoses made by the EMS relate to symptoms and complaints described by patients. This is due to limitations related to the diagnostic capabilities of the EMS. Therefore, most of the more than 14,000 different ICD-10 codes are not applicable in the prehospital emergency setting. In this situation, perhaps we should consider limiting the number of ICD-10 codes and making them dependent on the needs and diagnostic capabilities of the EMS.

The knowledge, skills, and experience of the members of the EMS have an impact on the accuracy and correctness of the diagnoses made. Similar roles are played by diagnostic tools on ambulance equipment, such as electrocardiographs. In many cases, making a more precise diagnosis is associated with the introduction and use in the ambulance service of additional diagnostic tools, such as ultrasound (currently being implemented in Poland), electronic patient medical history, or teleconsultation with a doctor specialist. At the same time, it should be remembered that the specifics of the work of the EMS, the lack of witnesses to the event, time pressure, etc., will not always allow a precise diagnosis to be made.

The limitation of the study is the prehospital diagnosis, which has the character of an initial examination of the patient. The use of modern technology in medicine undoubtedly contributes to making more accurate diagnoses. This is possible thanks to the appropriate preparation of the medical staff of the EMS.

## 5. Conclusions

The use of the ICD-10 classification has practical application in EMTs. It enables the identification of a disease or health problem, which facilitates further management and communication of medical personnel and analysis of medical data. The most common diagnoses were those from the ICD-10 group R00-R99-Symptoms, signs, and abnormal clinical and laboratory findings, not elsewhere classified (39.11%), which may be due to: knowledge of EMTs, limited diagnostic possibilities on board ambulances, the reliance of EMTs mainly on physical examination, and symptoms reported by patients and medical records. This requires further analysis. The groups of ICD-10 diagnoses upon which the diagnosis was based, and thus the reasons for EMT intervention, differ depending on the area of intervention (urban, rural), sex, and age of the patient and the method of completion of the activities by EMTs.

## Figures and Tables

**Table 1:** Interventions of emergency medical teams by ICD-10 diagnosis groups in 2017-2019.

Group ICD-10^*∗*^	Year 2017		Year 2018		Year 2019		Total	
	*n*	%	*n*	%	*n*	%	*n*	%
A00–B99	34	0.09	23	0.06	31	0.08	88	0.08
C00–D48	319	0.86	346	0.88	379	0.95	1,044	0.90
D50–D89	16	0.04	22	0.06	33	0.08	71	0.06
E00–E90	1,064	2.86	1,162	2.96	1,219	3.06	3,445	2.96
F00–F99	1,792	4.82	1,733	4.41	1,806	4.54	5,331	4.58
G00–G99	1,246	3.35	1,197	3.05	1,160	2.92	3,603	3.10
H00–H59	18	0.05	15	0.04	18	0.05	51	0.04
H60–H95	19	0.05	36	0.09	56	0.14	111	0.10
I00–I99	5,787	15.57	6,111	15.55	6,212	15.61	18,110	15.57
J00–J99	1,018	2.74	978	2.49	820	2.06	2,816	2.42
K00–K93	521	1.40	545	1.39	627	1.58	1,693	1.46
L00–L99	61	0.16	82	0.21	69	0.17	212	0.18
M00–M99	402	1.08	466	1.19	500	1.26	1,368	1.18
N00–N99	396	1.07	415	1.06	488	1.23	1,299	1.12
O00–O99	179	0.48	140	0.36	158	0.40	477	0.41
P00–P96	16	0.04	15	0.04	26	0.07	57	0.05
Q00–Q99	2	0.01	3	0.01	3	0.01	8	0.01
R00–R99	14,217	38.24	15,566	39.60	15,688	39.43	45,471	39.11
S00–T98	6,981	18.78	7,144	18.17	7,073	17.77	21,198	18.23
V01–Y98	1,970	5.30	2,079	5.29	2,093	5.26	6,142	5.28
Z00–Z99	1,120	3.01	1,230	3.13	1,333	3.35	3,683	3.17
Total	37,178	100.00	39,308	100.00	39,792	100.00	116,278	100.00

^*∗*^A00–B99 = Certain infectious and parasitic diseases; C00–D48 = Neoplasms; D50–D89 = Diseases of the blood and blood-forming organs and certain disorders involving the immune mechanism; E00–E90 = Endocrine, nutritional, and metabolic diseases; F00–F99 = Mental and behavioural disorders; G00–G99 = Diseases of the nervous system; H00–H59 = Diseases of the eye and eye appendages; H60–H95 = Diseases of the ear and mastoid process; I00–I99 = Diseases of the circulatory system; J00–J99 = Diseases of the respiratory system; K00–K93 = Diseases of the digestive system; L00–L99 = Diseases of the skin and subcutaneous tissue; M00–M99 = Diseases of the musculoskeletal system and connective tissue; N00–N99 = Diseases of the genitourinary system; O00–O99 = Pregnancy, childbirth, and the puerperium; P00–P96 = Certain conditions originating in the perinatal period; Q00–Q99 = Congenital malformations, deformations, and chromosomal abnormalities; R00–R99 = Symptoms, signs, and abnormal clinical and laboratory findings, not elsewhere classified; S00–T98 = Injury, poisoning, and certain other consequences of external causes; V01–Y98 = External causes of morbidity and mortality; Z00–Z99 = Factors influencing health status and contact with health services. Pearson Chi^2^ = 146.32, *df* = 40, *p* < 0.0001.

**Table 2:** Interventions of emergency medical teams according to ICD-10 diagnosis groups depending on the area of disposal.

Group ICD-10^*∗*^	Urban area		Rural area	
	*n*	%	*n*	%
A00–B99	34	0.09	54	0.07
C00–D48	338	0.87	706	0.91
D50–D89	18	0.05	53	0.07
E00–E90	1,009	2.59	2,436	3.15
F00–F99	1,942	4.98	3,389	4.39
G00–G99	1,298	3.33	2,305	2.98
H00–H59	21	0.05	30	0.04
H60–H95	28	0.07	83	0.11
I00–I99	5,850	15.00	12,260	15.87
J00–J99	742	1.90	2,074	2.68
K00–K93	494	1.27	1,199	1.55
L00–L99	75	0.19	137	0.18
M00–M99	519	1.33	849	1.10
N00–N99	393	1.01	906	1.17
O00–O99	180	0.46	297	0.38
P00–P96	12	0.03	45	0.06
Q00–Q99	4	0.01	4	0.01
R00–R99	14,772	37.87	30,699	39.73
S00–T98	7,351	18.85	13,847	17.92
V01–Y98	2,411	6.18	3,731	4.83
Z00–Z99	1,516	3.89	2,167	2.80
Total	39,007	100.00	77,271	100.00

^*∗*^see Table 1. Pearson Chi^2^ = 406.73, *df* = 20, *p* < 0.0001.

**Table 3:** Interventions of emergency medical teams by ICD-10 diagnosis groups depending on the patient's sex.

Group ICD-10^*∗*^	Male		Female	
	*n*	%	*n*	%
A00–B99	35	0.06	53	0.10
C00–D48	645	1.06	399	0.72
D50–D89	28	0.05	43	0.08
E00–E90	1,566	2.58	1,879	3.38
F00–F99	3,207	5.29	2,124	3.82
G00–G99	2,509	4.13	1,094	1.97
H00–H59	24	0.04	27	0.05
H60–H95	44	0.07	67	0.12
I00–I99	7,524	12.40	10,586	19.04
J00–J99	1,536	2.53	1,280	2.30
K00–K93	925	1.52	768	1.38
L00–L99	96	0.16	116	0.21
M00–M99	664	1.09	704	1.27
N00–N99	639	1.05	660	1.19
O00–O99	0	0.00	477	0.86
P00–P96	27	0.04	30	0.05
Q00–Q99	4	0.01	4	0.01
R00–R99	22,405	36.92	23,066	41.49
S00–T98	12,712	20.95	8,486	15.26
V01–Y98	4,211	6.94	1,931	3.47
Z00–Z99	1,877	3.09	1,806	3.25
Total	60,678	100,00	55,600	100,00

^*∗*^see Table 1. Pearson Chi^2^ = 3,394.57, *df* = 20, *p* < 0.0001.

**Table 4:** Interventions of emergency medical teams by ICD-10 diagnosis groups depending on the patient's age group.

Group ICD-10^*∗*^	Patient's age									
	0-14		15-29		30-49		50-69		70+	
	*n*	%	*n*	%	*n*	%	*n*	%	*n*	%
A00–B99	10	0.20	1	0.01	10	0.05	28	0.09	39	0.08
C00–D48	5	0.10	10	0.09	67	0.31	526	1.63	436	0.95
D50–D89	2	0.04	0	0.00	9	0.04	17	0.05	43	0.09
E00–E90	31	0.62	73	0.64	352	1.64	874	2.71	2,115	4.59
F00–F99	103	2.06	1,144	10.03	2,148	9.99	1,343	4.16	593	1.29
G00–G99	90	1.80	482	4.23	1,432	6.66	1,019	3.16	580	1.26
H00–H59	2	0.04	7	0.06	6	0.03	14	0.04	22	0.05
H60–H95	3	0.06	3	0.03	27	0.13	40	0.12	38	0.08
I00–I99	27	0.54	193	1.69	1,158	5.38	5,908	18.30	1,0824	23.49
J00–J99	238	4.77	42	0.37	156	0.73	641	1.99	1,739	3.77
K00–K93	39	0.78	64	0.56	255	1.19	477	1.48	858	1.86
L00–L99	10	0.20	19	0.17	26	0.12	67	0.21	90	0.20
M00–M99	9	0.18	115	1.01	379	1.76	390	1.21	475	1.03
N00–N99	7	0.14	158	1.39	322	1.50	374	1.16	438	0.95
O00–O99	0	0.00	244	2.14	233	1.08	0	0.00	0	0.00
P00–P96	55	1.10	2	0.02	0	0.00	0	0.00	0	0.00
Q00–Q99	6	0.12	0	0.00	0	0.00	2	0.01	0	0.00
R00–R99	1,828	36.65	3,337	29.25	6,946	32.29	1,2790	39.61	2,0570	44.65
S00–T98	1,823	36.55	3,699	32.43	5,143	23.91	5,191	16.08	5,342	11.59
V01–Y98	356	7.4	1,322	11.59	2,140	9.95	1,778	5.51	546	1.19
Z00–Z99	350	7.02	492	4.31	701	3.26	814	2.52	1,326	2.88
Total	4,988	100.00	1,1407	100.00	2,1510	100.00	3,2291	100.00	4,6074	100.00

^*∗*^see Table 1. Pearson Chi^2^ = 23,835.29, *df* = 80, *p* < 0.0001.

**Table 5:** Place of transfer of the patient and the ICD-10 group.

Group ICD-10^*∗*^	Place of transfer of the patient					
	Hospital		Remained		Death	
	*n*	%	*n*	%	*n*	%
A00–B99	65	0.08	23	0.08	0	0.00
C00–D48	681	0.79	336	1.23	27	1.02
D50–D89	69	0.08	2	0.01	0	0.00
E00–E90	2,131	2.47	1,313	4.81	1	0.04
F00–F99	3,880	4.50	1,451	5.31	0	0.00
G00–G99	2,115	2.45	1,487	5.44	1	0.04
H00–H59	41	0.05	10	0.04	0	0.00
H60–H95	79	0.09	32	0.12	0	0.00
I00–I99	14,087	16.32	3,430	12.56	593	22.44
J00–J99	2,327	2.70	484	1.77	5	0.19
K00–K93	1,389	1.61	301	1.10	3	0.11
L00–L99	135	0.16	77	0.28	0	0.00
M00–M99	601	0.70	767	2.81	0	0.00
N00–N99	872	1.01	426	1.56	1	0.04
O00–O99	472	0.55	5	0.02	0	0.00
P00–P96	50	0.06	7	0.03	0	0.00
Q00–Q99	7	0.01	1	0.00	0	0.00
R00–R99	34,527	40.00	9,068	33.19	1,876	70.98
S00–T98	18,830	21.82	2,347	8.59	21	0.79
V01–Y98	3,476	4.03	2,551	9.34	115	4.35
Z00–Z99	482	0.56	3,201	11.72	0	0.00
Total	86,316	100.00	27,319	100.00	2,643	100.00

^∗^see Table 1. Pearson Chi^2^ = 15669.11, *df* = 40, *p* < 0.0001.

## Data Availability

The dataset is available from the corresponding authors upon reasonable request and with permission from the Independent Public Healthcare Institution RM-MEDITRANS Ambulance and Sanitary Transport Station in Siedlce.
